# High-Fidelity Tissue Engineering of Patient-Specific Auricles for Reconstruction of Pediatric Microtia and Other Auricular Deformities

**DOI:** 10.1371/journal.pone.0056506

**Published:** 2013-02-20

**Authors:** Alyssa J. Reiffel, Concepcion Kafka, Karina A. Hernandez, Samantha Popa, Justin L. Perez, Sherry Zhou, Satadru Pramanik, Bryan N. Brown, Won Seuk Ryu, Lawrence J. Bonassar, Jason A. Spector

**Affiliations:** 1 Laboratory for Bioregenerative Medicine and Surgery, Division of Plastic Surgery, Weill Cornell Medical College, New York, New York, United States of America; 2 Department of Biomedical Engineering, Cornell University, Ithaca, New York, United States of America; 3 Department of Bioengineering, University of Pittsburgh, Pittsburgh, Pennsylvania, United States of America; The Ohio State University, United States of America

## Abstract

**Introduction:**

Autologous techniques for the reconstruction of pediatric microtia often result in suboptimal aesthetic outcomes and morbidity at the costal cartilage donor site. We therefore sought to combine digital photogrammetry with CAD/CAM techniques to develop collagen type I hydrogel scaffolds and their respective molds that would precisely mimic the normal anatomy of the *patient-specific* external ear as well as recapitulate the complex biomechanical properties of native auricular elastic cartilage while avoiding the morbidity of traditional autologous reconstructions.

**Methods:**

Three-dimensional structures of normal pediatric ears were digitized and converted to virtual solids for mold design. Image-based synthetic reconstructions of these ears were fabricated from collagen type I hydrogels. Half were seeded with bovine auricular chondrocytes. Cellular and acellular constructs were implanted subcutaneously in the dorsa of nude rats and harvested after 1 and 3 months.

**Results:**

Gross inspection revealed that acellular implants had significantly decreased in size by 1 month. Cellular constructs retained their contour/projection from the animals' dorsa, even after 3 months. Post-harvest weight of cellular constructs was significantly greater than that of acellular constructs after 1 and 3 months. Safranin O-staining revealed that cellular constructs demonstrated evidence of a self-assembled perichondrial layer and copious neocartilage deposition. Verhoeff staining of 1 month cellular constructs revealed *de novo* elastic cartilage deposition, which was even more extensive and robust after 3 months. The equilibrium modulus and hydraulic permeability of cellular constructs were not significantly different from native bovine auricular cartilage after 3 months.

**Conclusions:**

We have developed high-fidelity, biocompatible, patient-specific tissue-engineered constructs for auricular reconstruction which largely mimic the native auricle both biomechanically and histologically, even after an extended period of implantation. This strategy holds immense potential for durable patient-specific tissue-engineered anatomically proper auricular reconstructions in the future.

## Introduction

Microtia is reported to occur in 0.83 to 4.34 per 10,000 births, with higher incidences among males and those of Asian heritage [1]. Although the diagnosis of microtia encompasses a spectrum of phenotypes, ranging from “mild structural abnormalities to complete absence of the ear,” [1] even minor cases may incur psychological distress due to actual or perceived disfigurement and its effect on psychosocial functioning.

Autologous reconstruction techniques, in which costal cartilage is harvested, sculpted to recreate the three-dimensional structure of the auricle, and implanted under the periauricular skin, are the current gold standard for reconstruction of microtia [2] and other auricular deformities. Among the benefits of this approach are long-term stability [2], [3], [4], [5], a high degree of biocompatibility [6], the absence of antigenicity [3], and the potential for the graft to grow with the patient as he matures [2], [3], [4]. Despite these advantages, the use of autologous costal cartilage incurs numerous drawbacks, including a limited donor site supply [4], [5], [7] and significant donor site morbidity [2], [3], [4], [5], [7], [8], [9]. Other notable drawbacks associated with this approach are the immense difficulty inherent to sculpting an anatomically correct patient-specific auricular facsimile [3], [4], [7] and the inability for costal cartilage to adequately approximate the complex biomechanical properties of native auricular elastic cartilage [3], [9], all of which contribute to suboptimal aesthetic outcomes.

For these reasons, a tissue engineering-driven solution has long been sought for auricular reconstruction. Such a strategy entails the fabrication of a scaffold (either naturally-derived, synthetic, or a combination of the two) recapitulating the three-dimensional structure of the native external ear that could then be seeded with chondrocytes and subsequently implanted in the intended recipient. Over time, these grafted chondrocytes would secrete a new elastic cartilaginous matrix, thereby replacing the original scaffold while maintaining its contours. Indeed, execution of this strategy has been attempted previously and many clinically and commercially available synthetic polymers have been evaluated for this purpose. Benefits of their use include abundant supply, consistency in behavior, and the ability to be exactly sculpted into the desired configuration [2], [9]. However, as with all avascular synthetic materials, these polymers are limited by an increased susceptibility to infection and the risk of extrusion, as well as complications due to poor biocompatibility, host immune responses [2], [8], [9], potentially inflammatory degradation products, and unknown longevity and stability over time [2], [9].

Among the synthetic materials most commonly utilized for tissue-engineered auricular reconstruction are (FDA approved) polyglycolic acid (PGA) and polylactic acid (PLA) [4], [8], [9], polymers typically used together due to the cell compatibility of the former and the maintenance of strength over time of the latter. Despite their frequent use, however, these materials have been noted to incite unwanted inflammatory reactions [2], [3], attributed by some to the products of PLA degradation [6], [7]. In addition, high-density porous polyethylene (HDPP) scaffolds, while biocompatible and often used clinically for reconstructive purposes in other anatomic regions, are quite rigid unlike auricular native cartilage [3] and associated with increased rates of infection and extrusion [10], thus resulting in suboptimal reconstructions.

Synthetic (i.e., poloxamer) and naturally derived hydrogels (i.e., alginate, agarose, or fibrin) have similarly been evaluated as substrates for auricular tissue-engineered scaffolds as they are easily molded, potentially injectable, and “provide a hospitable three-dimensional support matrix” for cells contained within [3]. While biodegradable and used clinically, fibrin hydrogels are limited by their low tensile strength and poor surgical handling and are thus most often used as a coating for other, less-biocompatible materials to increase their cellular compatibility [4], [11].

Like fibrin, the extracellular matrix component collagen is abundant, biocompatible, and can be used in hydrogel form [12]. Indeed, collagen hydrogels have been utilized previously for cartilage tissue engineering applications, albeit with mixed results including the inability to independently maintain original cast dimensions without the use of an internal support [12], [13].

With the recent explosion of digital technology, computer-assisted design/computer-assisted manufacturing (CAD/CAM) techniques have emerged as a viable means of fabricating specific three-dimensional structures based upon virtual images. Despite the immense potential CAD/CAM approaches offer the field of tissue-engineered microtia reconstruction, few groups have effectively applied this technology towards auricular scaffold fabrication [7], [14]. Furthermore, digital acquisition of three-dimensional data has commonly relied on modalities such as computed tomography [7], which is expensive and imparts harmful ionizing radiation.

We therefore sought to combine digital photogrammetry with CAD/CAM techniques to develop high-density collagen type I hydrogel scaffolds and their respective molds that would precisely mimic the normal anatomy of the *patient-specific* external ear as well as recapitulate the complex biomechanical properties of native auricular elastic cartilage while avoiding the morbidity of traditional autologous reconstructions.

## Methods

### Ethics Statement

All animal care and experimental procedures were in compliance with the Guide for the Care and Use of Laboratory Animals [15] and were approved by the Weill Cornell Medical College Institutional Animal Care and Use Committee (protocol # 2011-0036). All efforts were made to minimize suffering.

### Isolation of chondrocytes

Bovine auricular chondrocytes were isolated as previously described [16]. Briefly, ears were obtained from freshly slaughtered 1–3 day old calves (Gold Medal Packing, Oriskany, NY). Auricular cartilage was sharply dissected from the surrounding skin and perichondrium under sterile conditions. Cartilage was diced into 1 mm^3^ pieces and digested overnight in 0.3% collagenase, 100 µg/mL penicillin, and 100 µg/mL streptomycin in Dulbecco's modified Eagle's medium (DMEM). The following day, the cells were filtered, washed, and counted.

### Construct design and mold fabrication

**Figure 1:**
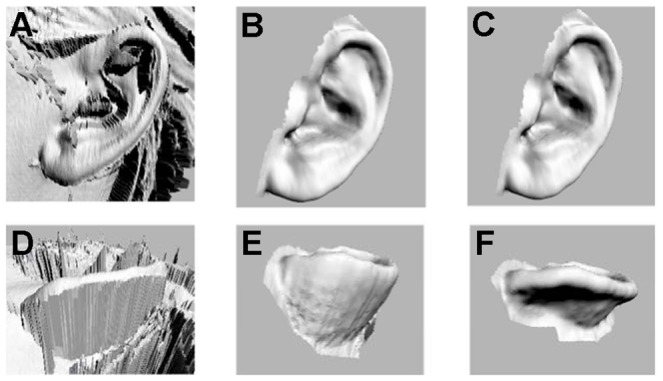
Digitization process for human ears. The anatomy of a 5 year-old female was scanned (*A*, *D*), processed to remove noise (*B*, *E*), and digitally sculpted to obtain the appropriate curvature for the anterior portion of the ear (*C*, *F*). Sagittal (*A–C*) and worm's-eye (*D–F*) views.

Molds for the generation of ear constructs were designed from digital images of human ears obtained from three-dimensional (3D) photogrammetry. High-resolution images of the ear of a five year-old female were obtained using a Cyberware Rapid 3D Digitizer (3030 Digitizer, Monterey, CA). By confining the scan to the region of the ear, approximately a 15° arc centered on the ear, the geometry of the auricle was obtained to within a resolution of 15 µm in approximately 60 seconds. These images were subsequently processed using PlyEdit software (Cyberware, Inc., Monterey, CA), first to remove digital noise and subsequently edited to produce an image with a continuous surface (Figure 1).

**Figure 2:**
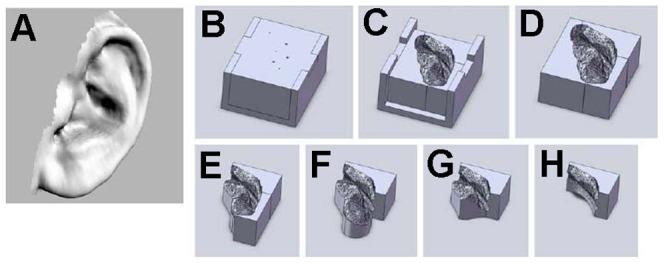
Mold design based on ear anatomy. The digital images of ears (*A*) were used to design 7-part molds (*B–H*) by embedding the solid images of the ear into virtual blocks.

These images were converted to stereolithography (.STL) files using Studio 4.0 (Geomagic, Morrisville, NC) and imported into SolidWorks (Dassault Systems Corp, Waltham, MA). The image of the 3D ear was embedded into a virtual block to cavity, which was used to design a 7-part mold using the part feature in SolidWorks (Figure 2). Each of the mold parts was printed out of acrylonitrile butadiene styrene (ABS) plastic using a Stratasys FDM 2000 3D printer (Eden Prairie, MN). Prior to use, all molds were sterilized by washing with Lysol® (Parsippany, NJ) followed by a 1-hour soak in 70% ethanol that was allowed to evaporate for 30 minutes in a sterile biological safety cabinet.

### Implant fabrication

Collagen for implant molding was extracted and reconstituted as previously described [17], [18]. Briefly, tendons were excised from 7–8 month-old mixed gender Sprague rat-tails and suspended in 0.1% acetic acid at 150 mL/gram of tendon for at least 48 hours at 4°C. The collagen solution was centrifuged for 90 minutes at 4500 RPM at 4°C. The clear supernatant was then collected and lyophilized, and the pellet was discarded. The collagen was reconstituted as a stock solution of 20 mg/mL collagen in 0.1% acetic acid.

The stock collagen solution was returned to pH 7.0 and maintained at 300 mOsm by mixing it with the appropriate volumes of 1N NaOH, 10× phosphate buffered saline (PBS), and 1× PBS as previously described [17], [19]. This collagen solution was immediately mixed with the cells and media and injected into ear molds using a syringe stop-cock system to obtain a final collagen concentration of 10 mg/mL and a final cell concentration of 25×10^6^ cells/mL. Separate acellular constructs were made through an identical process that did not involve suspension of cells in collagen. The molds were allowed to gel for 50 minutes at 37°C. After 50 minutes, the ear constructs were removed from the molds and cultured in media composed of DMEM, 10% fetal bovine serum, 100 µg/mL penicillin, 100 µg/mL streptomycin, 0.1 mM non-essential amino acids, 50 µg/mL ascorbate, and 0.4 mM L-proline. Samples were cultured in this media for 3–5 days until implantation. A total of 16 cell-seeded and 9 acellular samples were generated for this study. Two cell-seeded constructs were excluded from *ex vivo* analysis due to seroma formation.

### In vivo implantation

Ten-week old male athymic nude rats (RNU; Charles River, Wilmington MA) were used for *in vivo* studies. Animals were anesthetized via intraperitoneal injection of ketamine (80 mg/kg) and xylazine (8 mg/kg). After induction of anesthesia, the animal's dorsum was shaved, depilated, prepped with povidone iodine, and appropriately draped. All animals received a subcutaneous injection of buprenorphine (0.1 mg/kg) and an intraperitoneal injection of cefazolin (11 mg/kg) prior to any surgical manipulation. An incision was then made overlying the dorsum and the smallest subcutaneous pocket that would accommodate the implant (∼4×5 cm) was dissected in the loose subcutaneous areolar tissue. An acellular or cellular implant was then inserted and appropriately oriented. Incisions were closed with metallic wound clips and a sterile occlusive dressing was placed prior to recovery from anesthesia.

**Figure 3:**
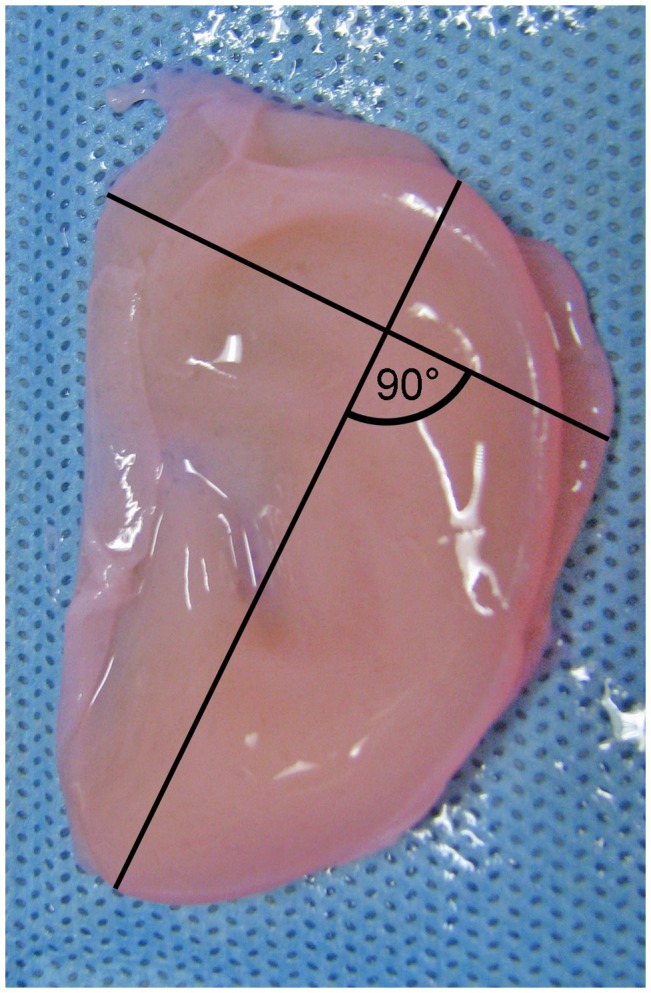
Schematic representation of length and width measurements. Construct length was measured along the lobule-helix axis. Construct width was defined as the largest dimension measured along an axis perpendicular to the lobule-helix axis.

Animals were sacrificed via CO_2_ asphyxiation and bilateral thoracotomy after 1 or 3 months. Constructs were harvested and their weights recorded. Construct length was measured along the lobule-helix axis. Construct width was defined as the largest dimension measured along an axis perpendicular to the lobule-helix axis (Figure 3). Half of each specimen was snap-frozen in liquid nitrogen for biomechanical analysis, while the remainder was fixed in 10% neutral buffered formalin for 48 hours prior to histologic analyses.

### Histologic analyses

The fixed portions of samples were dehydrated by sequential washes in ethanol, embedded in paraffin, cut into 5 µm sections, and stained with Safranin O/Fast green to assess proteoglycan distribution and Verhoeff's/Van Gieson to assess the presence of elastin fibers.

### Biomechanical analysis

Six mm×1 mm disks were cut from the central portion of frozen implants using dermal biopsy punches and thawed in PBS containing protease inhibitors. Disks were placed in a cylindrical confining chamber mounted in an ELF 3200 test frame (Enduratec, Eden Prarie, MN). Samples were compressed to 50% of their original height in 10×50 µm steps, with 5 minutes between steps to allow for full stress relaxation. Resultant stresses were recorded at 1 Hz and the temporal profiles of stress were fit to a poroelastic model of tissue behavior using custom MATLAB (MathWorks, Natick, MA) code to calculate the equilibrium modulus and hydraulic permeability [20], [21].

## Results

### Ex vivo gross analyses

Upon gross inspection of *in vivo* implants after 1 month, acellular implants had significantly decreased in size and lacked dorsal projection. In contrast, 1 month after implantation, cellular constructs retained their general contour visible through the thick skin of the rat, as well as their projection from the animal's dorsal surface. These findings were even more pronounced at 3 months: acellular specimens were barely visible through the animals' skin, while cellular constructs maintained their projection and surface characteristics.

**Figure 4:**
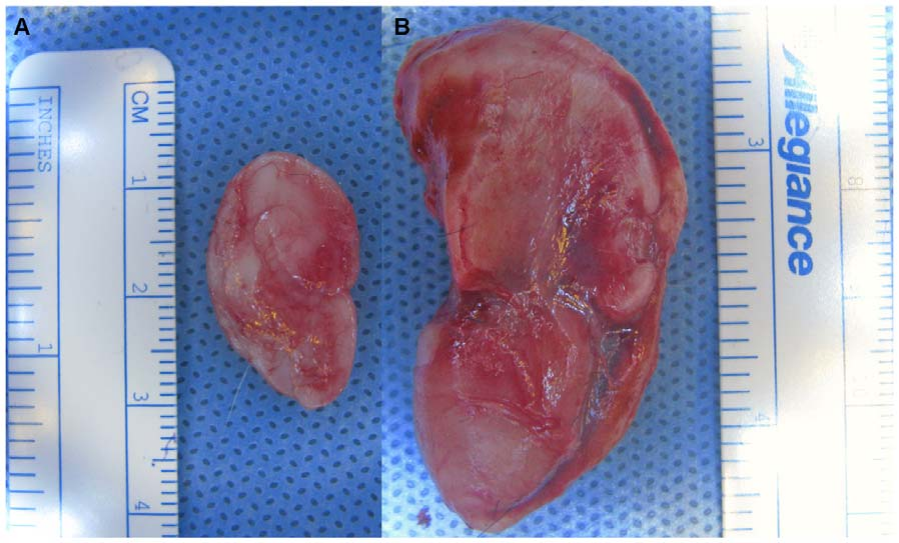
*Ex vivo* gross analysis. Three months after implantation, acellular implants (*A*) had decreased in size, whereas cellular constructs (*B*) retained their original anatomic fidelity.

*Ex vivo* analysis confirmed *in vivo* findings. One-month acellular constructs were wispy and amorphous, while cellular scaffolds maintained their tragus, lobule, helix, and antihelix features. This difference was even more apparent after 3 months: acellular implants had decreased in size, whereas cellular constructs retained their original anatomic fidelity (Figure 4).

**Figure 5:**
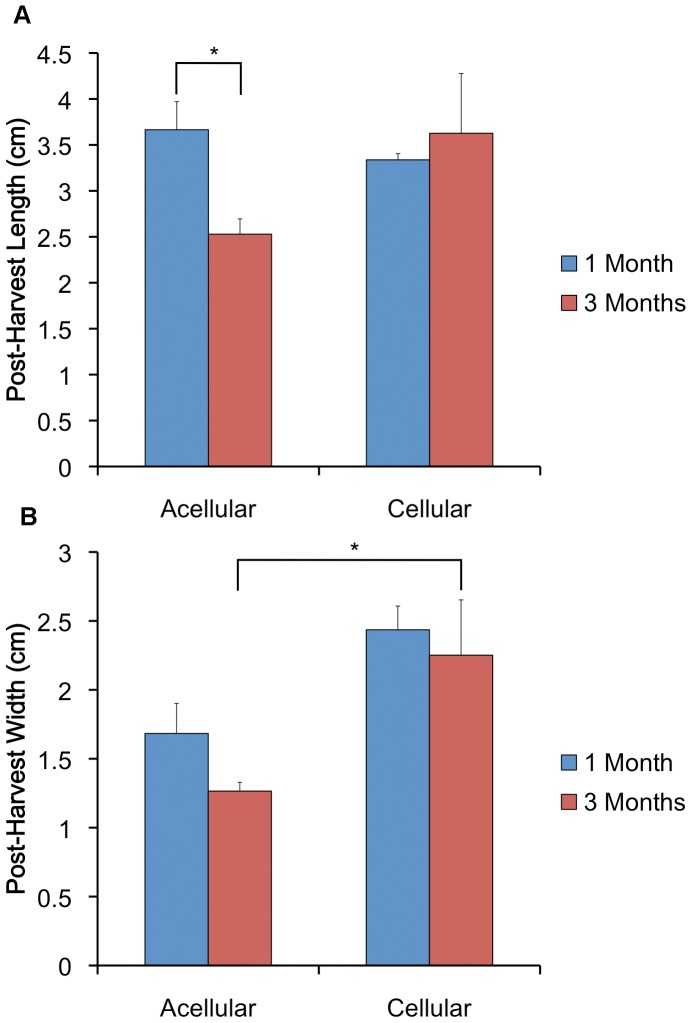
*Ex vivo* analysis of specimen length and width. (*A*) The length of acellular constructs harvested after 3 months was significantly less that that of constructs harvested after 1 month. In contrast, cellular construct length did not change over time. (*B*) Cellular construct width was significantly greater than acellular construct width at 3 months. * denotes p<0.05.

Post-harvest weight of cellular constructs was significantly greater than that of acellular constructs after 1 (4.17±0.17 g v. 0.80±0.07 g, p<1×10^−4^) and 3 (5.12±1.78 g v. 0.67±0.03 g, p = 0.021) months. The length of acellular constructs harvested after 3 months was significantly less that that of constructs harvested after 1 month (2.53±0.17 cm v. 3.67±0.30 cm, p = 0.009). In contrast, cellular construct length did not change over time (3.63±0.65 cm v. 3.34±0.07 cm at 3 months and 1 month, respectively). Lastly, cellular construct post-harvest width was significantly greater than acellular construct width at 3 months (2.25±0.90 cm v. 1.27±0.06 cm, p = 0.04) (Figure 5).

### Histologic analyses

**Figure 6:**
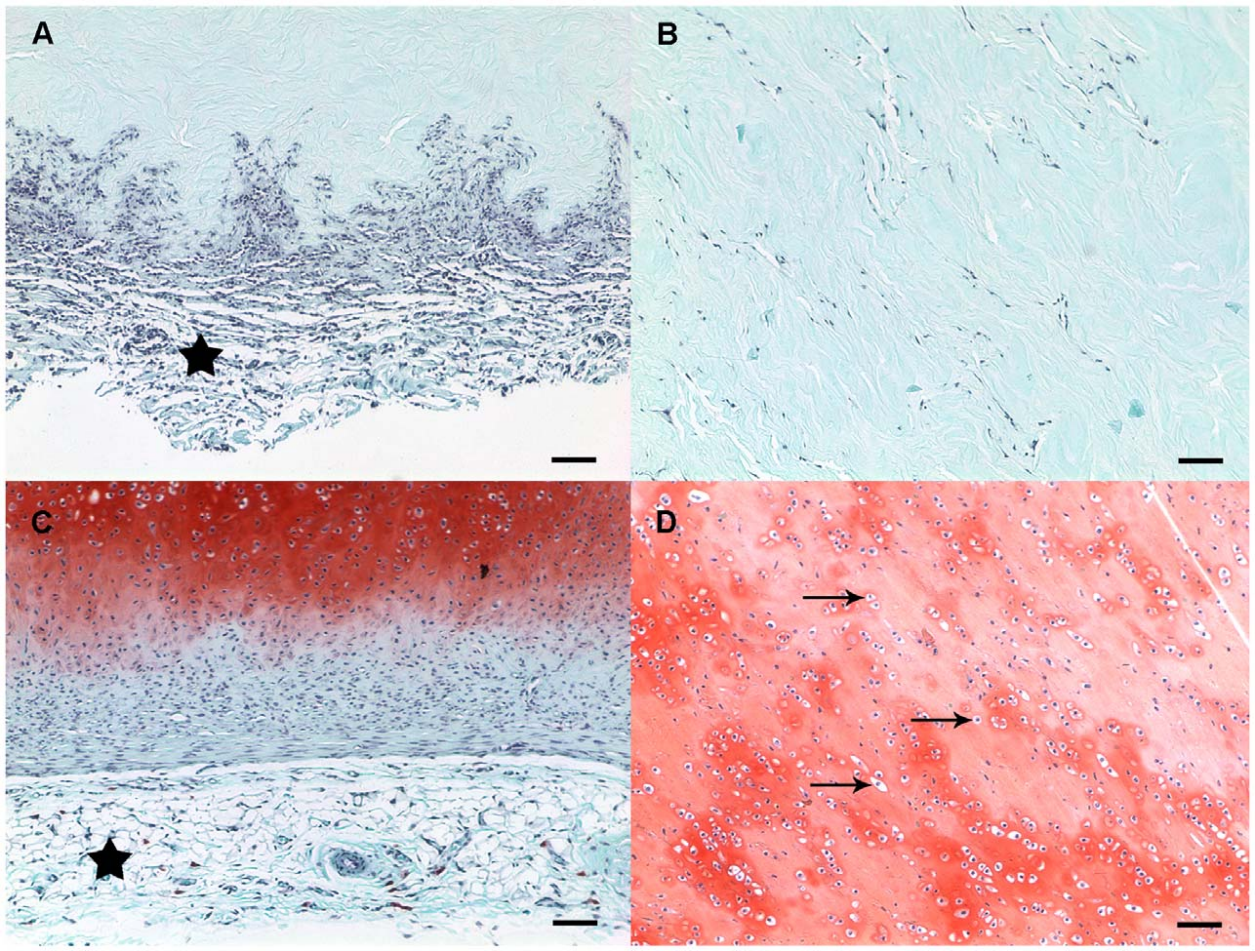
Safranin O staining of specimens harvested after 1 month. Acellular constructs (*A*) and cellular constructs (*C*) demonstrated evidence of a thin capsule containing spindle-shaped, fibroblast-appearing cells (*star*). Although the acellular constructs were invaded by mononuclear cells, there was no evidence of cartilage deposition (*B*). Cellular constructs demonstrated marked cartilage deposition by lacunar chondrocytes (*arrows*) throughout the construct (*B*, *D*). *Scale bar = 100 µm*.

Safranin O staining of acellular ears harvested after 1 month demonstrated histologic evidence of the formation of a thin capsule (not evident on gross inspection) by spindle-shaped fibroblast-appearing cells, as well as mononuclear cell invasion. However, even at the center of acellular specimens, there was no evidence of cartilage deposition. Cellular constructs harvested after 1 month demonstrated similar evidence of capsule formation and an even more robust infiltration of mononuclear cells. In addition, samples seeded with chondrocytes also demonstrated marked cartilage deposition by lacunar chondrocytes (Figure 6).

**Figure 7:**
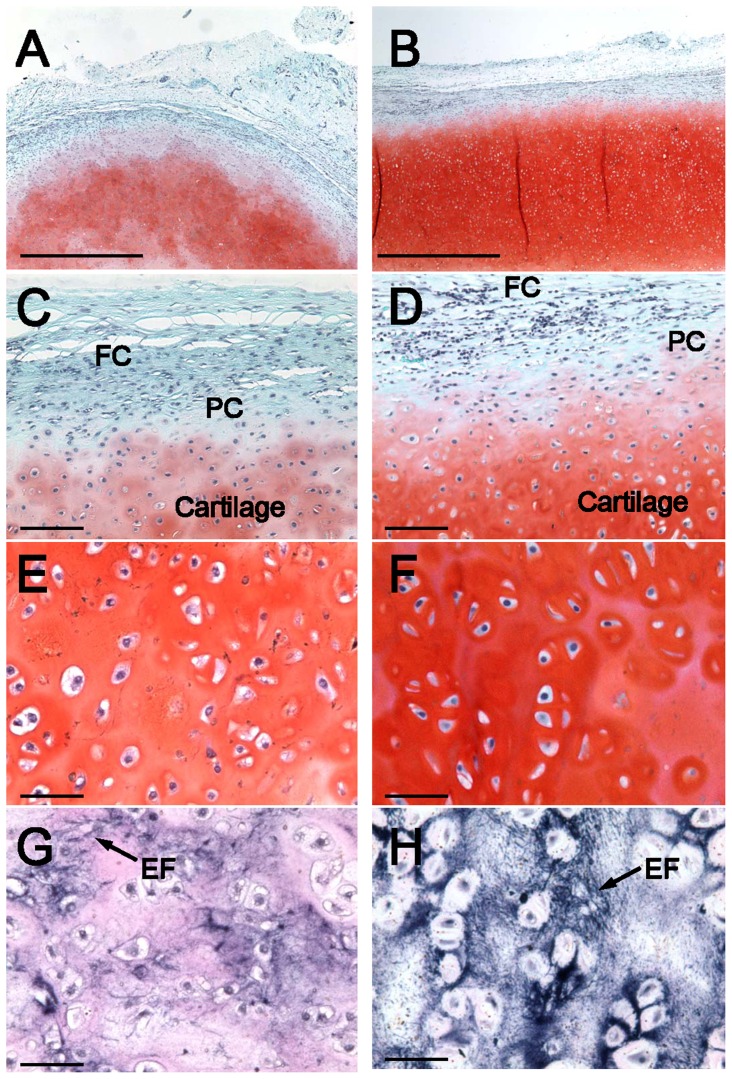
Histologic comparison of 1-month and 3-month samples by Safranin O and Verhoeff stains. Low magnification comparison between 1-month (*A*) and 3-month (*B*) Safranin O-stained sections (*A–F*) demonstrates more intense and uniform staining after 3 months (*scale bar = 1 mm*). Inspection of the edge of 1-month (*C*) and 3-month (*D*) samples shows a transition from the fibrous capsule (*FC*) to a perichondrial layer (*PC*) to cartilage (*scale bar = 100 µm*). High magnification comparison at 1-month (*E*) and 3-month (*F*) shows mature cartilage formation at both times (*scale bar = 50 µm*). Verhoeff's stain reveals the presence of elastin at both 1-month (*G*) and 3-months (*H*), with a more continuous network of elastin fibers after 3 months (*scale bar = 50 µm*).

Safranin O staining appeared to progress with time, with deeper and more uniform Safranin O staining occurring in cellular 3-month samples compared with 1-month samples (Figure 7). At both time points, cellular samples contained large areas of cartilage, several millimeters thick. Specimens appeared to contain a distinct layer between the newly formed cartilage and the surrounding fibrous capsule. This layer resembled a perichondrium, with cells that were more rounded than fibroblasts surrounded by matrix with minimal proteoglycan content. Deep within the cellular constructs, both 1- and 3-month samples had large regions of mature cartilage containing large rounded auricular chondrocytes.

At 1 month, samples contained focal areas with high elastin content as indicated by Verhoeff's stain. By 3 months, staining for elastin was more widespread and intense, with evidence of a large network of elastin fibers within the tissue.

Lastly, neither cellular nor acellular constructs appeared to elicit an inflammatory host response after 1 or 3 months, as indicated by the absence of polymorphonuclear cells or macrophages within or surrounding the constructs.

### Biomechanical analyses

**Figure 8:**
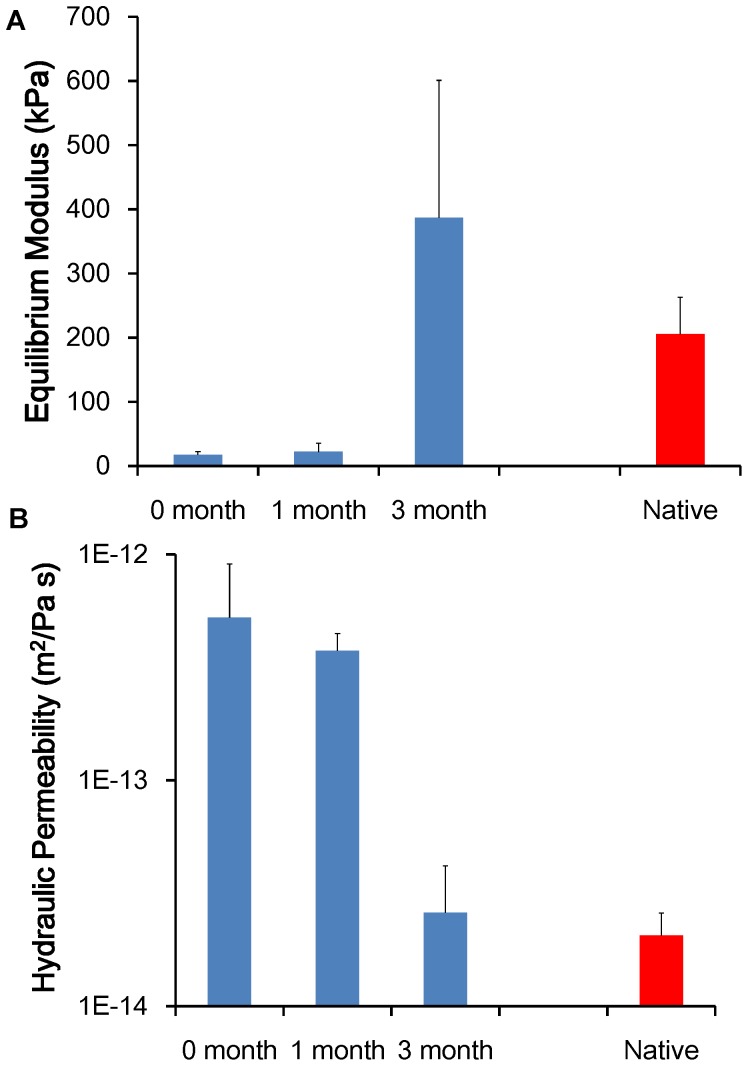
Equilibrium modulus and hydraulic permeability of tissue-engineered and native bovine auricular cartilage. Tissue-engineered auricular cartilage showed progressive improvement in mechanical properties with increasing time *in vivo*. The equilibrium modulus (*A*) and hydraulic permeability (*B*) of implants at 3 months were not statistically different from those of native bovine auricular cartilage. Data are displayed as mean+standard deviation for n = 4 for 0- and 1-month tissue-engineered samples, n = 5 for 3-month tissue-engineered samples, and n = 6 samples for native cartilage. * denotes p<0.05.

Tissue-engineered auricular cartilage showed progressive improvement in mechanical properties with increasing time *in vivo* (Figure 8). After 1 month, the equilibrium modulus was 3-fold higher (p<0.05) than prior to implantation and after 3 months was more than 30-fold higher (p<0.05) than pre-implantation. Likewise, hydraulic permeability was 5-fold lower (p<0.001) after 1 month and 70-fold lower at 3 months (p<0.001) compared with pre-implantation. The equilibrium modulus and hydraulic permeability of implants at 3 months were not statistically different from those of native bovine auricular cartilage.

## Discussion

Tissue-engineering approaches to auricular reconstruction offer the potential for the creation of more anatomically precise auricular facsimiles without incurring significant morbidity at the costal cartilage donor site, prolonged operative times to allow for shaping of the specimen, or the need for multiple operative procedures before the graft is suitable for elevation from the scalp [3].

However, like autologous reconstructions, current tissue-engineered auricular reconstructions are limited in their ability to accurately mimic normal auricular anatomy or biomechanical properties, let alone patient-specific anatomy. In this study, we have overcome these obstacles through the application of a novel method for construct design and fabrication. The digital photogrammetric acquisition of data utilized herein allows for high-resolution image capture without the risk of radiation exposure. Furthermore, as the image acquisition process is rapid (∼60 seconds), the need to subject children to restraints, sedatives or even general anesthesia to prevent movement is obviated. Lastly, constructs fabricated by these means represent exact mirror images of patients' contralateral normal ears and thus offer the potential for superior aesthetic outcomes surpassing even the most experienced hands. In the case of bilateral microtia, anatomically appropriate ears could be chosen from a “library” of patient images.

Historically, the failure of scaffolds to maintain their size is among the major obstacles of auricular tissue engineering [3], [12]. Inadequate cell seeding, incomplete replacement of the original scaffold by neocartilage deposition [2], [8], inability to withstand contractile forces *in vivo* [2], and “infiltration of noncartilaginous tissues” [8] have all been hypothesized to be causative factors. In addition, it is nearly impossible to evaluate how these factors contribute to scaffold deformation or degradation, as the majority of studies that investigate the potential for tissue engineering of elastic auricular cartilage utilize only sheets or fragments of material [5], [8], or ear-shaped constructs based upon molds from very small children (1–3 years) [6], [9], [11], [22], and not school-aged children (whose ears are ∼80% of their adult size).

In contrast to previous studies [2], [22], our cellular constructs successfully maintained not only their original dimensions but also their topography over time. We believe this successful preservation of their shape and size is attributable to the injectable, high-density collagen type I scaffold, which has not yet to our knowledge been described for the fabrication of full-sized, anatomically-correct facsimiles of the external ear (without the bolstering of an internal wire support). Not only did chondrocyte-containing specimens in this study demonstrate the deposition of copious elastic neocartilage highly similar to native human elastic with respect to both overall architecture and elastin content [23], but cellular specimens did not change appreciably in size during the interval of implantation. This suggests that the process of neocartilage deposition likely occurred at a rate similar to that of collagen degradation. Although the longest time point included in this study was 3 months, several earlier studies demonstrated construct shrinkage or deformation by this time [2], [4], [9], [22].

Rather than using type I collagen native to inelastic, weight-bearing tendons, it may seem more intuitive to use type II collagen as the basis for our construct bulk. However, the use of type II collagen in our injection molding system is problematic, as its solubility is insufficient to yield the high-density (i.e., 15–20 mg/ml) hydrogels needed to retain dimensional stability after molding. Indeed, studies using type II collagen hydrogels as a scaffold for chondrocytes report concentrations in the range of 1–3 mg/ml [24], [25], which is inadequate for our purposes. Furthermore, a large number of studies report excellent results using type I collagen as a scaffold material for cartilage tissue engineering. Such studies report that chondrocytes seeded within these materials produce tissues that contain predominantly type II collagen [26].

As such, cellular constructs in the current study demonstrated the deposition of elastic neocartilage, as evidenced by characteristic Safranin O and Verhoeff staining. While many studies offer evidence of neocartilage production by chondrocytes in lacunae [2], [4], [5], [6], [8], [9], [11], [12], [13], [22], [27], few demonstrate the presence of elastin within specimens or utilized chondrocytes of auricular origin [2], [8], [12], [13], [22]. This distinction is important, as few chondrocytes (only those in the external ear, nasal septum, epiglottis, and corniculate and cuneiform cartilages) specifically elaborate elastic cartilage. Furthermore, given differences in location, development, and local signaling milieu, it cannot be assumed that elastin-producing chondrocytes of non-auricular origin generate elastic cartilage identical to that found in the external ear. It is for these reasons that we believe auricular chondrocytes represent the optimal cell source for future tissue-engineered auricular reconstructions.

The native ear is frequently loaded and can experience a range of loading modes, including tension, compression, and bending. As a result, studies have evaluated the tensile [28], compressive [16], [29], [30], [31], and bending [32] properties of tissue-engineered ear cartilage. The success of our approach to ear cartilage tissue engineering is highlighted by the mechanical properties of the tissue produced. By 3 months, the equilibrium modulus (a measure of tissue stiffness) and the hydraulic permeability (a measure of the ease with which fluid can flow through the tissue) were similar to those of bovine auricular cartilage as well as human nasal septal cartilage [33]. The analogous data for human auricular cartilage are not readily available in the literature. Furthermore, relatively few studies have similarly evaluated the mechanical performance of tissue-engineered ear cartilage. In addition, we chose to evaluate the compressive properties of cartilage using confined compression testing, as this is the most reliable method to obtain the poroelastic material properties of cartilage.

Only one other study to date [16] has demonstrated the formation of ear cartilage that is stable in a long-term animal model with material properties comparable to native ear cartilage. This previous study used a similar injection molding technique with alginate as the scaffold material and required up to 6 months following implantation in sheep to form fully mechanically competent implants [20]. In contrast, the current study using injection molded collagen implants showed similar results after only 3 months *in vivo*.

Despite its initial success, our technique would require modifications prior to translation to human subjects. An immunocompromised host was utilized in this study, and therefore the constructs implanted were not necessarily subject to the same degree of scaffold degradation, vascularization, or host cell invasion as would be seen in immunocompetent models. The immune response to both cellular and acellular scaffolds therefore necessitates evaluation in an immunocompetent host, as one could theoretically be mounted against either non-autologous collagen or cellular inhabitants. In addition, the chondrocytes utilized in this study were of bovine origin. However, to facilitate translation to the clinical realm, the identical methodology could be applied using patient-specific chondrocytes derived from the patient's own microtic ear remnant, or potentially even autologous bone marrow- or adipose-derived mesenchymal stem cells, or some combination thereof. This substitution would eliminate the immune response to non-autologous cells within the construct. Non-autologous collagen (i.e., bovine and porcine) is already commonly utilized for clinical purposes, is well tolerated as such, and therefore is of less concern as a potential antigenic stimulus. Lastly, although it is unlikely that construct degradation would occur beyond 3 months, verification of construct stability over a longer implantation interval (i.e., 6–12 months) must be performed.

## Conclusions

Digital photogrammetry was successfully combined with CAD/CAM and tissue injection molding techniques to create high-fidelity, biocompatible, patient-specific tissue-engineered constructs for auricular reconstruction without the use of imaging modalities that incur ionizing radiation. We believe that our cellular constructs' appropriate biomechanical properties and maintenance of volume, shape and topographical characteristics over time can be attributed in part to their type I collagen hydrogel composition, which allows for the optimal rates of chondrocyte growth, matrix resorption, and the *in vivo* deposition of elastic cartilage. Although this strategy holds immense potential for tissue-engineered auricular reconstructions, construct evolution over a longer implantation interval (i.e., 6–12 months) and ultimately, use of patient-specific chondrocytes and/or mesenchymal stem cells must be evaluated prior to translation of this technology to the clinical realm.
